# Consumption of antibiotics in Brazil - an analysis of sales data between 2014 and 2019

**DOI:** 10.1186/s13756-024-01412-6

**Published:** 2024-06-09

**Authors:** Luciane Cruz Lopes, Fabiane R. Motter, Mônica Da Luz Carvalho-Soares

**Affiliations:** 1 Pharmaceutical Science Graduate Course, University of Sorocaba, Rodovia Raposo Tavares, Km 92.5, Sorocaba, São Paulo, Brazil; 2 Brazilian Health Regulatory Agency, Brasilia, Brazil

**Keywords:** Antibiotic consumption, Antimicrobial resistance, Defined daily doses, AWaRe, Time-series study

## Abstract

**Background:**

Antibiotic consumption is a driver for the increase of antimicrobial resistance. The objective of this study is to analyze variations in antibiotic consumption and its appropriate use in Brazil from 2014 to 2019.

**Methods:**

We conducted a time series study using the surveillance information system database (SNGPC) from the Brazilian Health Regulatory Agency. Antimicrobials sold in retail pharmacies were evaluated. All antimicrobials recorded for systemic use identified by the active ingredient were eligible. Compounded products and formulations for topic use (dermatological, gynecological, and eye/ear treatments) were excluded. The number of defined daily doses (DDDs)/1,000 inhabitants/day for each antibiotic was attributed. The number of DDDs per 1,000 inhabitants per day (DDIs) was used as a proxy for consumption. Results were stratified by regions and the average annual percentage change in the whole period studied was estimated. We used the WHO Access, Watch, and Reserve (AWaRe) framework to categorize antimicrobial drugs.

**Results:**

An overall increase of 30% in consumption from 2014 to 2019 was observed in all Brazilian regions. Amoxicillin, azithromycin and cephalexin were the antimicrobials more consumed, with the Southeast region responsible for more than 50% of the antibiotic utilization. Among all antimicrobials analyzed 45.0% were classified as watch group in all Brazilian regions.

**Conclusion:**

We observed a significant increase in antibiotics consumption from 2014 to 2019 in Brazil restricted to the Northeast and Central West regions. Almost half of the antibiotics consumed in Brazil were classified as watch group, highlighting the importance to promote rational use in this country.

**Supplementary Information:**

The online version contains supplementary material available at 10.1186/s13756-024-01412-6.

## Introduction

The widespread use of antibiotics plays a major role concerning antimicrobial resistance across the world leading to challenges in health management [1]. However, knowledge gaps still exist regarding the extent and appropriateness of their use in low- and middle-income countries(LMIC) [2].

An assessment of 71 countries demonstrated an increase of 35% in antibiotics consumption between 2000 and 2010, with Russia, India, China, South Africa, and Brazil accounting for 76% of the increase [3]. Beyond the growth in consumption, LMIC also demonstrate an inappropriate use of antibiotics. Between 2000 and 2015, Klein et al. [4] demonstrated through the World Health Organization (WHO) Access, Watch, and Reserve (AWaRe) antibiotic classification framework [5], an increase in Watch antibiotic consumption, especially in these countries.

The Brazilian Health Regulatory Agency (Anvisa) initial Regulation nº 20/2011^1^ established criteria for prescribing, dispensing, controlling, packaging, and labelling antimicrobial drugs [6]. Starting in 2011, all antibiotics were restricted access, and mandatory reports on dispensing started to be requested. Brazil has sought to control the over-the-counter sale of these drugs through the National Controlled Products Management System (SNGPC). However, few studies have demonstrated the trends of consumption over time using SNGPC data. From 2013 to 2016, an overall growth of 18% in antibiotic consumption was observed in Brazil, ranging from 4 to 85% across the Brazilian regions [7–9]. Related to the appropriateness of use, no study has used the AWaRe antibiotic classification framework [5] to demonstrate the patterns of consumption in Brazilian regions.

This study aims to describe the trends of antibiotic consumption in Brazil and its regions using sales data of drugstores and pharmacies provided by the SNGPC. We will use the AWaRe Classification to demonstrate the appropriateness of use in Brazilian regions.

## Methodology

### Study design

This is a time-series study from 2014 to 2019 in Brazil.

### Data source

We used data from SNGPC the surveillance information system that records prescriptions subject to special control. The recording of antimicrobials prescribed by physicians, veterinary and dentists sailed in retail in private pharmacies and drugstores is mandatory in Brazil since 2013 [6]. Data records include month, year, and geographic location (federal unit) where the products were sold, prescriber’s professional order; active ingredient and salt; dose form and strengths, and amount (number of units sold). The SNGPC system does not record information on products dispensed in hospitals, in the public health system or distributed by non-governmental organizations. SNGPC only *records movements of entry (Purchase and transfers) and exits (sales, transfers, and losses) of medications sold in private pharmacies and drugstores in the countr.y*.

### Data management, measurement, and variables

All antimicrobials for systemic use identified according to the active ingredient (including the salt) and dose form/strengths were analyzed in this study. We excluded records coded as compounded products, products for topic use as dermatological, gynecological, and antimicrobials for eye and ear treatments. Also, we did not include products prescribed by veterinarians’ doctors. Even though the data refers to medication administered outside hospitals, it can represent up to 80% of total consumption in many countries [8]. The list of variables and definitions is provided in the supplemental material (Additional File 1, Table S1).

We used the Anatomical Therapeutic Chemical (ATC) classification system for categorizing the medications within the following pharmacological subgroup: tetracyclines (J01A), amphenicols (J01B) penicillins (J01C), cephalosporins(J01D), sulfonamides and trimethoprim (J01E), macrolides, lincosamides and streptogramins (J01F), aminoglycoside antibacterials (J01G), quinolone antibacterials (J01M) and other antibacterials (J01X) (which includes metronidazole).

### Volume sales of antimicrobials

We analyzed the sales data of the antimicrobials dispensed in Brazil to verify the total number sales for each medication from 2014 to 2019. Annual sales for all antibiotics were aggregated to provide one value for total per region in 2014 and 2019.

### Antimicrobials consumption

We converted sales expressed in kilograms into the number of Defined Daily Doses (DDDs) using the ATC System (ATC/DDD, 2020). We also calculated the number of DDDs per 1000 inhabitants per day (DDI), as a proxy for consumption. We adopted population estimates from the IBGE (Brazilian Institute of Geography and Statistics) reported for the inter-census years (2014–2019). The 27 Brazilian Federated Units (26 States and the Federal District) were further grouped into five regions (North, Northeast, South-East, South, and Central-West).

The annual DDI was calculated based on the number of packs/bottles dispensed using the following formula:

[annual sale amount (mg) × 1000] / [DDD (mg) × 365 × study population]

Where Annual sale amount = [amount of active ingredient in each pharmaceutical form (mg) × number of pharmaceutical forms per unit × units (packs/bottles) sold].

Monthly consumption was calculated using the same formula, substituting the 365 days of the year with the number of days in the respective month.

### WHO Access, Watch, and Reserve (AWaRe) antibiotic classification framework

We adopted the categorization proposed by WHO [5] to analyze the appropriate access to antibiotics in Brazil. This classification takes into account the impact of different antibiotics and antibiotic classes on antimicrobial resistance, to emphasize the importance of their appropriate use and categorized antimicrobial drugs into access, watch, and reserve group (Box 1).

**Box 1:** WHO categories of antibiotics – descriptions

Group	Definition
Access group	First- and second-choice antibiotics that should be widely available in all countries. They should be affordable and quality assured.
Watch group	First- and second-choice antibiotics that only should be used for a specific, limited number of indications due to higher resistance potential.
Reserve group	Last resort antibiotics that should be used only when other antibiotics have failed or for infections of multi-resistant bacteria.

All antibiotics were assigned to the AWaRe categories. The combinations of antibiotics that were not identified to the AWaRe categories had each antibiotic evaluated separately.

DDI of antibiotics in the AWaRe categories was calculated as the sum of the DDIs of the antibiotic belonging to each category and presented by year and Brazilian regions. Percentages of antibiotics in the AWaRe categories were calculated by applying the proportion of antibiotic consumption (DDI) in each group in 2019.

### Statistical analysis

We estimated the DDI trends using join point regression [10] or every region and year by using the Join point Regression Program, Version 4.9.0.0 (Statistical Research and Applications Branch, National Cancer Institute).

Briefly, by using DDIs as inputs, the method identifies the year(s) when a trend change is produced, calculates the annual percentage change (APC) between trend-change points, and it also estimates the average annual percentage change (AAPC) in the whole period studied. The overall analysis was conducted using Stata (https://www.stata.com) version 12.

### Ethics statement

All data were supplied aggregated at the country level without individual-level information. Therefore, ethical approval for this analysis was not required.

## Results

### Volume of sales products, 2014–2019

A total of 310,779,762 sales were identified during the study period. The total antimicrobials sales increased by 31.2%, from 44,964,792 in 2014 to 59,319,550 in 2019. Analysis by region showed that the highest number of sales were observed in the Southeast (23,219,570 sales in 2014 and 30,087,600 sales in 2019) and the in South region (8,584,818 sales in 2014 and 11,451,390 sales in 2019) in both years. The Southeast region was responsible for more than 50% of the antimicrobials sales, while the north region was responsible for about 5,0% of the antimicrobials sales. The southeast region corresponds to 41.8% of the Brazilian population, followed by the northeast region 26.9% and the South region 14.7%. The North and Central West regions contribute only 8% each to the total Brazilian population. Nevertheless, the largest increase of sales was observed in the Central-west region (55.4%), following the South region (33.4%) (Additional file 2, Table S2).

### DDD, ATC and WHO Access, Watch, and Reserve (AWaRe) antibiotic classification framework

Table 1 shows 53 antimicrobials for systemic use in humans marketed in Brazil according to pharmacological subgroups (3rd level of ATC) and substance (5th level of ATC). Amoxicillin, azithromycin and cefalexin are the antimicrobials with the largest sales volumes between 2014 and 2019.

**Table 1:** Profile of antimicrobials for systemic use sold in Brazil from 2014 to 2019, brazilian health regulatory agency (ANVISA)

Pharmacological subgroup	ATC Code	Substance	Number of Sales	Commercial Units Sold	Consumed tons	Commercial presentations Available in Brazil	DDD (g)	WHO AWaRe classification
Aminoglycoside Antibacterials	J01GB03	Gentamicin (IV)	156,016	355,958	0.07	28	0.24	Access
	J01GB06	Amikacin	3,523	69,511	0.03	11	1.00	Access
	J01GB01	Tobramycin (IV)	121	5,620	< 0.01	02	0.24	Watch
		Tobramycin (powder/solution)	66	86	< 0.01	04	0.11/0.30	Watch
Amphenicols	J01BA02	Thiamphenicol	107,333	121,665	0.96	02	1.50	Access
	J01BA01	Chloramphenicol	25,202	33,961	0.34	46	3.00	Access
Beta-Lactam Antibacterials, Penicillins	J01CA04	Amoxicillin	61,646,665	68,025,441	670.00	200	1.50	Access
	J01CR02	Amoxicillin/clavulanic Acid	47,900,000	56,800,000	528.90	42	1.50	Access
	J01CE08	Benzathine benzylpenicillin	2,176,780	4,022,629	15.50	59	3.60	Access
	J01CA01	Ampicillin	1,481,210	2,136,560	15.20	56	2.00	Accsess
	J01CR02	Amoxicillin/sulbactam	444,903	487,820	5.02	15	1.50	Not recommended
	J01CE02	Phenoxymethylpenicillin	67,446	106,354	0.40	105	2.00	Access
	J01CE09	Procaine benzylpenicillin	1,188	3,686	< 0.01	75	0.60	Access
	J01CE01	Benzylpenicillin	1,088	3,358	< 0.01	101	3.60	Access
	J01CR05	Piperacillin/ /tazobactam	117	809	< 0.01	12	14.00	Watch
	J01CF04	Oxacilin	46	374	< 0.01	1	2.00	Access
	J01CR01	Ampicillin/sulbactam	12	143	< 0.01	95	6.00	Access
Drugs for treatment of tuberculosis	J04AB02	Rifampicin	144,169	355,347	0.64	6	0.6	Watch
Macrolides, Lincosamides And Streptogramins	J01FA10	Azithromycin	50,140,000	63,300,000	163.00	132	0.30	Watch
	J01FA09	Clarithromycin	5,938,185	6,755,171	34.20	80	0.50	Watch
	J01FF01	Clindamycin (Oral/IV)	2,553,785	4,272,715	21.10	25	1.20/ 1.80	Access
	J01FF02	Lincomycin	367,748	926,513	0.50	04	1.80	Watch
	J01FA01	Erythromycin	512,335	623,599	4.40	28	1.00	Watch
	J01FA06	Roxithromycin	17	67	< 0.01	01	0.30	Watch
Other Antibacterials	J01XD01	Metronidazole (IV/ Oral)	7,819,066	9,573,397	74.80	48	1.50/2.00	Access
	J01XX08	Linezolid	1,296	2,075	< 0.01	05	1.20	Reserve
	J01XA02	Teicoplanin (IV)	293	1,344	< 0.01	11	0.40	Watch
	J01XB02	Polymyxin B (IV)	17	52	< 0.01	04	0.15	Reserve
Other Beta-Lactam Antibacterials	J01DB01	Cefalexin	38,500,000	72,600,000	3,460.0	102	2.00	Access
	J01DD04	Ceftriaxone	4,620,641	13,000,000	27.70	131	2.00	Watch
	J01DB05	Cefadroxil	5,351,102	9,530,943	39.40	30	2.00	Access
	J01DC04	Cefaclor	3,563,485	4,132,973	25.20	40	1.00	Watch
	J01DB03	Cefalotin	1006	4207	0.26	16	4.00	Access
	J01DH02	Meropenem	963	1,747	0.02	04	3.00	Reserve
	J01DE01	Cefepime	330	3298	< 0.01	28	4.00	Watch
	J01DD02	Ceftazidime	182	2158	< 0.01	13	4.00	Watch
	J01DB04	Cefazolin	175	632	< 0.01	11	3.00	Access
	J01DC01	Cefoxitin	145	386	< 0.01	03	6.00	Watch
	J01DF01	Aztreonam	86	210	< 0.01	02	4.00	Reserve
	J01DC02	Cefuroxime	5	53	< 0.01	02	0.50	Watch
	J01DD01	Cefotaxime	8	69	< 0.01	05	4.00	Watch
Quinolone Antibacterials	J01MA02	Ciprofloxacin (Oral/ IV)	31,545,477	36,110,587	239.14	142	1.00/0.80	Watch
	J01MA12	Levofloxacin	21,389,060	23,886,710	93.94	70	0.24	Watch
	J01MA06	Norfloxacin	7,551,935	8,271,836	44.60	55	0.80	Watch
	J01MA14	Moxifloxacin	1,554,924	1,801,077	4.33	19	0.40	Watch
	J01MA15	Gemifloxacin (Oral)	96,580	104,562	0.19	39	0.32	Watch
	J01MA01	Ofloxacin	1	1	< 0.01	1		
Sulfonamides and Trimethoprim	J01EE01	Sulfamethoxazole/trimethoprim	11,400,000	15,400,000	106.0	105	2.00	Access
Tetracyclines	J01AA02	Doxycycline	2,856,431	4,462,703	6.84	23	0.10	Access
	J01AA07	Tetracycline	766,525	2,793,014	38.50	60	1.00	Access
	J01AA08	Minocycline	169,793	207,017	0.61	38	0.20	Watch
	J01AA06	Oxytetracycline	9,189	23,017	0.09	04	1.00	Watch
	J01AA12	Tigecycline	14	28	< 0.01	01	0.10	Reserve

Of total of antimicrobials analyzed, 24 (45.0%) are classified as watch group, 5 (9.4%) reserve group and 1 (1.9%) is not recommended according to WHO AWaRe Classification. Table 2 shows antimicrobials consumption by WHO AWaRe category during 2014–2019.

**Table 2:** DDI according to WHO Access, and Watch categories, stratified by region from 2014 to 2019

Regions/WHO	2014	2015	2016	2017	2018	2019
**Access group**						
North	1.22	1.29	1.35	1.45	1.47	1.59
Northeast	1.20	1.30	1.37	1.51	1.56	1.70
Southeast	2.53	2.72	2.95	3.27	3.19	3.41
South	2.93	3.18	3.49	3.64	3.63	3.88
Central-West	2.25	2.44	2.68	3.12	3.18	3.61
**Watch group**						
North	1.15	1.25	1.22	1.28	1.32	1.32
Northeast	1.23	1.35	1.38	1.52	1.57	1.56
Southeast	2.89	3.11	3.31	3.47	3.27	3.22
South	3.62	3.72	4.09	4.12	4.01	3.85
Central-West	2.44	2.52	2.55	2.88	2.89	3.10

**Fig. 1:**
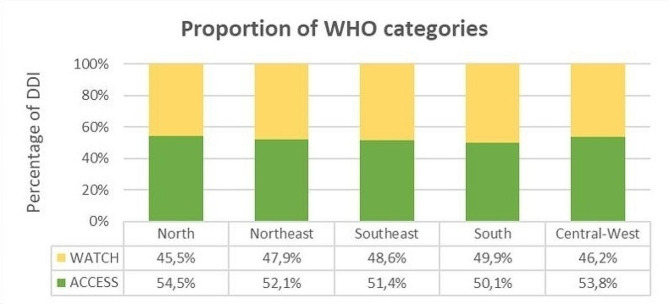
Proportion of antibiotic consumption according to WHO Access and Watch categories by region, 2019. Legends: DDI = Defined Daily Doses/1000population per day; **All estimates were calculated from January to December 2019

More than half of consumption of antibiotic consumption consisted of Access antibiotics in all Brazilian regions, Fig. 1. The proportion of “Access” to antibiotics was higher in North (54,5%) and Central-West (53.8%) regions while the proportion of “Watch” was higher in South (49.9%) and Southeast regions (48.6%).

### Trends in consumptions, 2014–2019

South region was the largest consumer of antimicrobials in all the years analyzed while the North region was lowest consumer. The Analysis by pharmacological subgroups was presented in Additional files 3–9 (Figures S1- S7). The most consumed groups were J01C (β-lactam penicillins), J01F (macrolides, lincosamides, and streptogramins), and J01M (Quinolone Antibacterials).

Azitromicin, Amoxicilin and Amoxicilin plus clavulanate, ciprofloxacin and cefalexin were the most consumed antimicrobial in this study. When we analysed the 72 monthly DDI of these antimicrobials, Table 3, there were statistically significant increases in total consumption of this antimicrobial over the study period in both Northeast and Central west regions. In relation to other antimicrobials analyzed, we found significantly increased of monthly DDIs between 2014 and 2019 in all regions in Brazil.

**Table 3:** DDI according to WHO Access, and Watch categories, stratified by region from 2014 to 2019

Substance	Region	2014 Mean (95% CI)	2015 Mean (95% CI)	2016 Mean (95% CI)	2017 Mean (95%CI)	2018 Mean (95%CI)	2019 Mean (95% CI)	*P* value	2014–2019 Trends
Azitromicin (J01FA10)	Brazil	1.10 (0.98–1.22)	1.21 (1.06–1.35)	1.29 (1.13–1.43)	1.32 (1.17–1.47)	1.22 (1.10–1.35)	1.21 (1.07–1.35)	0.097	↔
	Noth	0.47 (0.44–0.50)	0.51 (0.46–0.57)	0.49 (0.46–0.52)	0.49 (0.45–0.53)	0.49 (0.44–0.53)	0.49 (0.46–0.53)	0.907	↔
	Notheast	0.54 (0.50–0.57)	0.60 (0.56–0.64)	0.62 (0.59–0.66)	0.68 (0.62–0.73)	0.68 (0.63–0.72)	0.67 (0.62–0.72)	**< 0.001**	↑
	South-East	1.31 (1.16–1.45)	1.48 (1.27–1.68)	1.61 (1.42–1.79)	1.63 (1.44–1.81)	1.47 (1.32–1.61)	1.46 (1.27–1.64)	0.119	↔
	South	1.96 (1.68–2.24)	2.03 (1.71–2.36)	2.21 (1.84–2.58)	2.18 (1.81–2.54)	2.02 (1.66–2.38)	1.93 (1.63–2.23)	0.771	↔
	Central-West	1.09 (0.98–1.20)	1.10 (0.98–1.23)	1.07 (0.99–1.16)	1.22 (1.10–1.34)	1.15 (1.06–1.23)	1.28 (1.16–1.41)	**0.002**	↑
Amoxicilin (J01CA04)	Brazil	0.86 (0.78–0.94)	0.90 (0.83–0.98)	0.94 (0.86–1.02)	1.03 (0.95–1.12)	1.00 (0.92–1.07)	1.02 (0.95–1.09)	**< 0.001**	↑
	Noth	0.43 (0.40–0.46)	0.45 (0.41–0.48)	0.45 (0.42–0.47)	0.49 (0.46–0.53)	0.49 (0.46–0.52)	0.52 (0.49–0.54)	**< 0.001**	↑
	Notheast	0.45 (0.41–0.48)	0.47 (0.44–0.51)	0.48 (0.45–0.50)	0.53 (0.49-0.56)	0.52 (0.48–0.55)	0.53 (0.50–0.57)	**< 0.001**	↑
	South-East	1.14 (1.02–1.25)	1.18 (1.07–1.28)	1.23 (1.11–1.34)	1.35 (1.24–1.46)	1.27 (1.18–1.37)	1.28 (1.19–1.38)	**0.001**	↑
	South	1.13 (1.02–1.25)	1.20 (1.07–1.33)	1.31 (1.17–1.44)	1.39 (1.22–1.55)	1.35 (1.19–1.50)	1.39 (1.24–1.53)	**< 0.001**	↑
	Central -West	0.79 (0.72–0.85)	0.85 (0.78–0.91)	0.89 (0.83–0.95)	1.08 (1.00-1.16)	1.07 (1.00-1.13)	1.16 (1.10–1.23)	**< 0.001**	↑
Amoxicillin + clavulanate (J01CR02)	Brazil	0.58 (0,50-0.65)	0.64 (0.57–0.71)	0.74 (0.66–0.82)	0.86 (0.76–0.96)	0.89 (0.80–0.98)	1.04 (0.92–1.15)	**< 0.001**	↑
	Noth	0.22 (0.20–0.25)	0.24 (0.22–0.27)	0.25 (0.23–0.27)	0.29 (0.27–0.31)	0.32 (0.28–0.35)	0.36 (0.33–0.39)	**< 0.001**	↑
	Notheast	0.31 (0.28–0.34)	0.33 (0.31–0.35)	0.35 (0.33–0.37)	0.41 (0.39–0.45)	0.46 (0.42–0.50)	0.56 (0.50–0.59)	**< 0.001**	↑
	South-East	0.77 (0.66–0.88)	0.85 (0.74–0.96)	1.00 (0.88–1.11)	1.16 (1.01–1.31)	1.16 (1.04–1.27)	1.34 (1.18–1.49)	**< 0.001**	↑
	South	0.72 (0.60–0.84)	0.83 (0.70–0.95)	1.01 (0.85–1.16)	1.11 (0.94–1.28)	1.18 (0.97–1.38)	1.35 (1.14–1.55)	**< 0.001**	↑
	Central-West	0.63 (0.56–0.70)	0.70 (0.62–0.77)	0.78 (0.71–0.84)	0.97 (0.88–1.05)	1.04 (0.94–1.13)	1.30 (1.16–1.44)	**< 0.001**	↑
Ciprofloxacin (J01MA02)	Brazil	0.47 (0.45–0.49)	0.52 (0.50–0.53)	0.54 (0.52–0.55)	0.58 (0.56–0.59)	0.58 (0.56–0.60)	0.53 (0.51–0.55)	**< 0.001**	↑
	Noth	0.32 (0.30–0.33)	0.35 (0.34–0.36)	0.35 (0.34–0.36)	0.37 (0.36–0.38)	0.38 (0.37–0.40)	0.36 (0.35–0.37)	**< 0.001**	↑
	Notheast	0.30 (0.29–0.32)	0.34 (0.32–0.35)	0.35 (0.34–0.35)	0.37 (0.36–0.38)	0.37 (0.36–0.39)	0.35 (0.33–0.36)	**< 0.001**	↑
	South-East	0.57 (0.54–0.59)	0.62 (0.60–0.64)	0.64 (0.62–0.66)	0.70 (0.67–0.72)	0.69 (0.66–0.72)	0.62 (0.59–0.65)	**< 0.001**	↑
	South	0.59 (0.57–0.62)	0.64 (0.62–0.67)	0.67 (0.64–0.69)	0.71 (0.69–0.73)	0.73 (0.70–0.76)	0.66 (0.63–0.69)	**< 0.001**	↑
	Central-West	0.50 (0.48–0.52)	0.56 (0.54–0.58)	0.60 (0.57–0.62)	0.65 (0.63–0.67)	0.67 (0.64–0.71)	0.65 (0.62–0.67)	**< 0.001**	↑
	Brazil	0.35 (0.34–0.36)	0.35 (0.34–0.36)	0.35 (0.34–0.36)	0.35 (0.34–0.36)	0.35 (0.34–0.36)	0.35 (0.34–0.36)	**< 0.001**	↑
Cefalexin (J01DB01)	Noth	0.25 (0.24–0.26)	0.27 (0.26–0.27)	0.27 (0.27–0.28)	0.28 (0.27–0.29)	0.28 (0.27–0.29)	0.28 (0.27–0.29)	**< 0.001**	↑
	Notheast	0.22 (0.21–0.24)	0.25 (0.24–0.26)	0.26 (0.25–0.27)	0.27 (0.26–0.28)	0.26 (0.26–0.28)	0.27 (0.26–0.28)	**< 0.001**	↑
	South-East	0.43 (0.42–0.44)	0.45 (0.43–0.47)	0.50 (0.49–0.52)	0.49 (0.47–0.51)	0.45 (0.44–0.48)	0.47 (0.45–0.48)	**< 0.001**	↑
	South	0.41 (0.40–0.43)	0.44 (0.42–0.47)	0.50 (0.48–0.52)	0.50 (0.48–0.52)	0.48 (0.46–0.50)	0.49 (0.47–0.51)	**< 0.001**	↑
	Central-West	0.33 (0.32–0.35)	0.37 (0.36–0.39)	0.44 (0.43–0.45)	0.44 (0.42–0.46)	0.43 (0.41–0.44)	0.45 (0.44–0.47)	**< 0.001**	↑

## Discussion

### Main finding

Visual inspection of the consumption trends is sufficient to identify an increase (∼ 30%) in prescriptions dispensed from 2014 to 2019, in all Brazilian regions. Amoxicillin, azithromycin and cefalexin are the antimicrobials with the largest sales volumes. The Southeast region was responsible for more than 50% of the antimicrobials sales. Northeast and Central-west regions have statistically significant increases (*p* < 0.01) in total consumption DDIs of azithromycin, amoxicillin and amoxicillin plus clavulanate, ciprofloxacin and cefalexin. There were significant percentage of (10%) of antibiotics consumption from reserve group and not recommended group according to WHO AWaRe classification.

### Comparison with literature and previous studies

Previous studies using data from the IMS Health (IQVA) van Boeckel (2000–2010) [3] Moura et al. (2008–2012) [11] and Neves e Castro et al. (2013–2016) [9] pointed out that amoxicillin, cephalexin, and azithromycin were the most consumed drugs and had increases in the respective periods of analysis, consistent with our findings. However, the growth in consumption of amoxicillin plus clavulanate, ciprofloxacin and levofloxacin in this analysis period is noteworthy. Ciprofloxacin and levofloxacin are classified as antibiotics in the Watch group (antibiotics that should only be used for a specific, limited number of indications due to higher resistance potential [12]. Levofloxacin and ciprofloxacin have very similar activity profiles. Levofloxacin is approved by Anvisa for use in upper and lower respiratory tract infections, complicated and uncomplicated skin and subcutaneous tissue infections, urinary tract infections and osteomyelitis [12].Ciprofloxacin is Anvisa approved for the treatment of urinary tract infections, sexually transmitted infections, lower respiratory tract infections, inhalation anthrax, plague, and salmonellosis, acute bacterial exacerbation of chronic bronchitis [12]. Respiratory fluoroquinolones offer widespread microbiological coverage, have a suitable dosing schedule, and have the ability to switch from parenteral to oral therapy. However, excessive use of respiratory fluoroquinolones can induce subsequent emergence of multidrug-resistant organisms among treated patients, as has also been observed with β-lactams [13]. Ciprofloxacin and fluoroquinolones in general should be therapeutic options (not first indication) due to the risk of adverse effects, as recommended by the European Medicines Agency (EMA) [14], followed by Brazilian protocol [13]. EMA’s safety committee highlighted that fluoroquinolone antibiotics, given by any route is restricted due to the risk of disabling, long-lasting and potentially irreversible side effects [14]. A study conducted in Brazil in 2012 showed 10% of E. coli and 19% of K. pneumoniae resistant to ciprofloxacin isolated from urine samples [15]. A study [16], also carried out in Brazil, has shown 35% of E. coli resistant to ciprofloxacin, drawing attention to the significant increase in the rate of resistance to this drug. The growth in ciprofloxacin and levofloxacin consumption, mainly in the community, provides opportunities for interventions to control their excessive use. Hospital antimicrobial stewardship strategies that usually focus on changes to antimicrobial use practices have also confirmed their value in delivering clinical and economic benefits, with reductions in length of stay a crucial driver of cost savings [17]. Community interventions can be developed, building on existing evidence that communication skills training and changing patient expectations to receive antibiotics can lead to significant reductions in antibiotic prescriptions [18].

Even though the main antibiotics consumed are the same in all states, the proportions of dispensed volumes are very different, evidencing the unequal use to antibiotics. The southeast region, the richest in the country, has the largest economic center with the highest HDI (0.753) of Brazil and was responsible for more than 50% of the antimicrobials sales both in 2014 and in 2019, in contrast to the northern region that presents the lowest HDI (0.667) is the lowest percentage (5.0%) of the antimicrobials sales over these years. Northeast and South had similar sales percentages (∼ 19%). These numbers suggest a relationship between antibiotic distribution and population density since the Southeast region concentrates 42% of the Brazilian population and is the largest consumer. However, the second largest population (28%) in the country, which is in the Northeast region, consumed 42% less than the South region, which has half the population of the Northeast, indicating that factors such as income and maybe availability must play a role greater in this composition distribution.

Neves e Castro [9] found similar results, but consumption in the Northeast at that time (2013–2016) was 38% less than in the South region. On the other hand, the largest increase in sales was observed in the Central-west region (55.4%), which went from ∼ 8% of total sales in 2014 to ∼ 9% in 2019. Currently, 7.4% of the Brazilian population lives in the Central-West region of the country and in recent decades there have been federal incentives for economic growth, which seems to have influenced the purchase of antibiotic drugs. This fact was also observed in studies [8, 19] who evaluated the consumption of antibiotics in high-income countries and in emerging economies, showing that consumption rates per person increase rapidly in emerging economies, a fact observed in the central-west region of Brazil. Increased income is one of the main drivers of the increase in antibiotic consumption in low- and middle-income regions [19]. Thus, although antibiotic consumption rates in most low- and middle-income countries (LMIC) remain below the overall rate when compared to high income countries (HIC), these LMIC rates are expected to increase over time, and possibly exceed, antibiotic consumption rates in HIC, in part due to the higher burden of infectious disease in low- and middle-income countries.

Antibiotic drugs were introduced in high-income countries after mortality rates from infectious diseases had already decreased after the effect of water treatment, improved sanitation, and immunization, whereas, in many low-income and lower-middle-income countries, antibiotics are used as a substitute for public Health measures. Moreover, the prevalence of bacterial resistance generally correlated with the magnitude of antibiotic consumption in different Organization for Economic Cooperation and Development (OECD) countries [20].

Access, Watch and Reserve (AWaRe) antibiotics, and concordance with the monitoring indicator that 60% of total consumption should be Access agents [4, 5, 21]were assessed by region and in total period of analysis 2014–2019.

In all regions, there was a significant consumption of antibiotics from the watch group (45–49%). The southern region had the highest consumption of antibiotics in the Watch group over all years, followed by the South-East and Central-West regions. Quinolones (Ciprofloxacin, levofloxacin, norfloxacin), Macrolides (azithromycin) and Other Beta-Lactam Antibacterials (ceftriaxone) showed high sales volumes in all regions studied. Only quinolones did not show a downward trend or stabilization in consumption in the Midwest region. The same pattern was observed for macrolides. However, other beta-lactams show consumption trends still increasing in all regions. High levels of consumption of Watch agents are an obvious target for interventions including a review of clinical guidelines and prescribing algorithms.

There was a significant percentage of (10%) of antibiotics consumed from the Reserve group and not recommended group (Amoxicillin/sulbactam) according to WHO AWaRe classification. Amoxicillin/sulbactam, Tigecycline, aztreonam, meropenem, polymyxin, and linezolid indicated for hospital use should be considered antibiotics of last resort, which should be tailored to highly specific patients and settings, when all alternatives have failed or are not suitable were sold by pharmacies in the community. According to Brazilian legislation, these antibiotics are restricted for sale to hospitals. We cannot understand why they are being sold by pharmacies in the community. We do not know whether measures to check these sales deviations were investigated by Anvisa.

Effective strategies for better utilization of antimicrobials are crucial to address the growing concern of antimicrobial resistance. It is essential to emphasize the importance of implementing clear guidelines for the use of antimicrobials, promoting antimicrobial stewardship programs in healthcare facilities, and educating healthcare professionals and the general public about the appropriate use of these medicines. Furthermore, surveillance of antimicrobial use and resistance are important components of a comprehensive strategy to ensure the responsible and effective use of antimicrobials.Amoxicillin (*n* = 200), Ciprofloxacin (*n* = 142), Azithromycin (*n* = 132), Ceftriaxone (*n* = 131), Sulfamethoxazole/Trimethoprim (*n* = 105), Phenoxymethylpenicillin (*n* = 105), Cephalexin (*n* = 102), benzylpenicillin (*n* = 101), Ampicillin/sulbactam (*n* = 95) are the drugs with the highest number of commercial presentations available in the Brazilian market. Most products are generic. It is worth mentioning that there is an imbalance in the proportion of products of some antibiotics in relation to others, which may favor an imbalance between supply and demand within the different types of products. Of course, the newer antibiotics are still under patent law and few labs produce them. However, the exaggerated offer of some products can favor non-rational use, stimulated sales and the lack of competition due to the low number of presentations of other products can generate market monopoly and access difficulties.

### Strenght and limitations

This study has some limitations. Sales of medicines in pharmacies and drugstores do not guarantee that the medicines purchased were effectively dispensed to and used by patients.

Also, when calculating the monthly DDD/TID rates, we had to assume that the population was stable throughout the entire year, as we only had annual population data. In addition, factors other than restricting over-the-counter sales may have influenced, for example, changes in the economy, demographic factors, and the influence of the pharmaceutical industry. These considerations require further study. This study is not accounting for antibiotic consumption by SUS, which is substantial. Thus, the results are biased in showing consumption associated only with antibiotic purchases.

This study is that it only includes data from the community, drugstores and pharmacies, unrelated to data from hospitals. Thus, we were able to portray the use of antibiotics outside hospital environments. The important strength of this study is that we could analysis the Nationwide data, covering six years of antibiotic sale.

The data presented here provide a Brazilian perspective on rates and patterns of antibiotic consumption, by AWaRe category, between the years 2014 and 2019, the period leading up to the COVID-19 pandemic. In the absence of information linked to the indication of antibiotic use, the WHO AWaRe classification allows for a more detailed analysis of aggregated data and opportunities for Stewardship activities [21]. The use of the aWaRe classification in our study makes it possible to easily understand a simple metric of antibiotic use by prescribers and policy makers. AWaRe groups are now explicitly linked to the WHO Pathogen Priority List, directing specific actions for observed deviations.

### Health policy implications

Antimicrobial resistance (AMR) is a global challenge and a threat to health and the environment. COVID-19 has demonstrated the critical links between humans, animals and the environmental ecosystem. The pandemic has highlighted the health authority’s responsibility to prevent, prepare for and respond to emerging and re-emerging AMR. Our findings point to the need for a publicly funded international surveillance initiative to provide policy makers with evidence-based information on global, regional, and national rates and trends in antibiotic consumption.

The ANVISA has been reviewing and updating its National Action Plan for AMR since 2018, but some adjustments still need to be made. This is an ongoing process. This analysis can contribute to improving the quality of information made available by this data source and strengthen national and regional surveillance systems through better data management and implementation of data-driven practices and in this way contribute to the Global Surveillance System. Use of WHO Antimicrobial Resistance.

## Conclusion

We identified an increase (∼ 30%) in prescriptions dispensed from 2014 to 2019, in all Brazilian regions. The percentage of antibiotic use in the access group is below the 60% recommended by the WHO. In all Brazilian regions there was high consumption of antibiotics from the Watch group (∼ 50%). Our findings highlight opportunities for interventions/programs that promote rational use through coordinated efforts between regions and the federal government should be a priority. The monitoring antibiotic consumption and use patterns complements antimicrobial resistance surveillance by providing an understanding of the types and quantities of antibiotics being used, which can then inform policies, regulations, and interventions to optimize antibiotic use. Antibiotic consumption monitoring may also prompt review of surveillance health regulations and of procurement and supply chains of medicines as part of overall pharmaceutical systems strengthening.

### Electronic supplementary material

Below is the link to the electronic supplementary material.

Supplementary Material 1

Supplementary Material 2

Supplementary Material 3

Supplementary Material 4

Supplementary Material 5

Supplementary Material 6

Supplementary Material 7

Supplementary Material 8

Supplementary Material 9

## Data Availability

The datasets supporting the conclusions of this article are included within the article and its additional files.

## Footnotes

1 This RDC 20/2011 was revoked to RDC 471/2021 that defines the same criteria for prescription, dispensing, packaging, control and labeling of antimicrobials subject to control by this resolution. The rules for dispensing and controlling antimicrobials were not changed. BRAZIL. Anvisa. Agência Nacional de Vigilância Sanitária. RESOLUÇÃO – RDC Nº 471, DE 23 DE FEVEREIRO DE 2021. Access: 13/fev-2024.

## References

[1] Jiang T, Chen XS. Outcome impacts due to Pathogen-Specific Antimicrobial Resistance: a narrative review of published literature. Int J Environ Res Public Health. 2020;17(4). 10.3390/ijerph17041395 PMC7068360 32098182

[2] Founou RC, Founou LL, Essack SY (2017). Clinical and economic impact of antibiotic resistance in developing countries: a systematic review and meta-analysis. PLoS ONE.

[3] Sulis G, Adam P, Nafade V, Gore G, Daniels B, Daftary A (2020). Antibiotic prescription practices in primary care in low- and middle-income countries: a systematic review and meta-analysis. PLoS Med.

[4] Klein EY, Van Boeckel TP, Martinez EM, Pant S, Gandra S, Levin SA et al. Global increase and geographic convergence in antibiotic consumption between 2000 and 2015. Proceedings of the National Academy of Sciences of the United States of America https://org/101073/pnas1717295115. 2018;115(15):E3463–70. 10.1073/pnas.1717295115 PMC5899442 29581252

[5] WHO. The 2019 WHO AWaRe classification of antibiotics for evaluation and monitoring of use. https://www.who.int/news/item/01-10-2019-who-releases-the-2019-aware-classification-antibiotics. 2019.

[6] BRAZIL, Anvisa. Agência Nacional de Vigilância Sanitária. RESOLUÇÃO – RDC Nº 20, DE 5 DE MAIO DE 2011. Access: 08/jan-2023. 2011.

[7] Carias CM, Vieira FS, Giordano CV, Zucchi P (2011). Exceptional circumstance drug dispensing: history and expenditures of the Brazilian Ministry of Health. Rev Saude Publica.

[8] Van Boeckel TP, Gandra S, Ashok A, Caudron Q, Grenfell BT, Levin SA (2014). Global antibiotic consumption 2000 to 2010: an analysis of national pharmaceutical sales data. Lancet Infect Dis.

[9] Neves e Castro PB, da Silva Rodrigues DA, Roeser HMP, da Fonseca Santiago A (2020). Cássia Franco Afonso RJ. Antibiotic consumption in developing countries defies global commitments: an overview on Brazilian growth in consumption. Environ Sci Pollut Res.

[10] Dragomirescu I, Llorca J, Gómez-Acebo I, Dierssen-Sotos T (2019). A join point regression analysis of trends in mortality due to osteoporosis in Spain. Sci Rep.

[11] Moura ML, Boszczowski I, Mortari N, Barrozo LV, Neto FC, Lobo RD (2015). The impact of restricting Over-the-counter sales of antimicrobial drugs: Preliminary Analysis of National Data. Medicine.

[12] BRAZIL, Anvisa. Bulario. Bulario Available in: https://consultasanvisagovbr/#/bulario Acesso em: Jan/2023. 2023.

[13] Corrêa RA, AN C, Lundgren F, Michelim L (2018). e. 2018 recommendations for the management of community acquired pneumonia. J Bras Pneumol.

[14] EMA. Fluoroquinolone antibiotics: reminder of measures to reduce the risk of long-lasting, disabling and potentially irreversible side effects. European Medicines Agency Avilable on: https://www.emaeuropaeu/en/news/fluoroquinolone-antibiotics-reminder-measures-reduce-risk-long-lasting-disabling-potentially-irreversible-side-effects Accessed in: April 28th, 2024. 2023.

[15] Santana TCFS, Pereira EMM, Monteiro SG, Carmo MS, Turri RJG, Figueiredo PMS (2012). Prevalence and bacterial resistance in urinary tract infections in São Luis, MA, Brazil in the period from 2005 to 2008. Rev patol trop.

[16] D’Addazio LB, Moraes SR (2016). Microrganismos isolados de infecção do trato urinário Da comunidade. Revista De Saúde.

[17] Nathwani D, Varghese D, Stephens J, Ansari W, Martin S, Charbonneau C (2019). Value of hospital antimicrobial stewardship programs [ASPs]: a systematic review. Antimicrob Resist Infect Control.

[18] Strumann C, Steinhaeuser J, Emcke T, Sönnichsen A, Goetz K (2020). Communication training and the prescribing pattern of antibiotic prescription in primary health care. PLoS ONE.

[19] Klein EY, Milkowska-Shibata M, Tseng KK, Sharland M, Gandra S, Pulcini C (2021). Assessment of WHO antibiotic consumption and access targets in 76 countries, 2000-15: an analysis of pharmaceutical sales data. Lancet Infect Dis.

[20] Thomas MG, Smith AJ, Tilyard M (2014). Rising antimicrobial resistance: a strong reason to reduce excessive antimicrobial consumption in New Zealand. N Z Med J.

[21] Sharland M, Gandra S, Huttner B, Moja L, Pulcini C, Zeng M (2019). Encouraging AWaRe-ness and discouraging inappropriate antibiotic use-the new 2019 essential Medicines List becomes a global antibiotic stewardship tool. Lancet Infect Dis.

