# Supplementary figures and images for: Consumption of antibiotics in Brazil - an analysis of sales data between 2014 and 2019
Source: Antimicrob Resist Infect Control. 2024 Jun 9;13:60. doi: 10.1186/s13756-024-01412-6 (PMC11163732; doi:10.1186/s13756-024-01412-6)

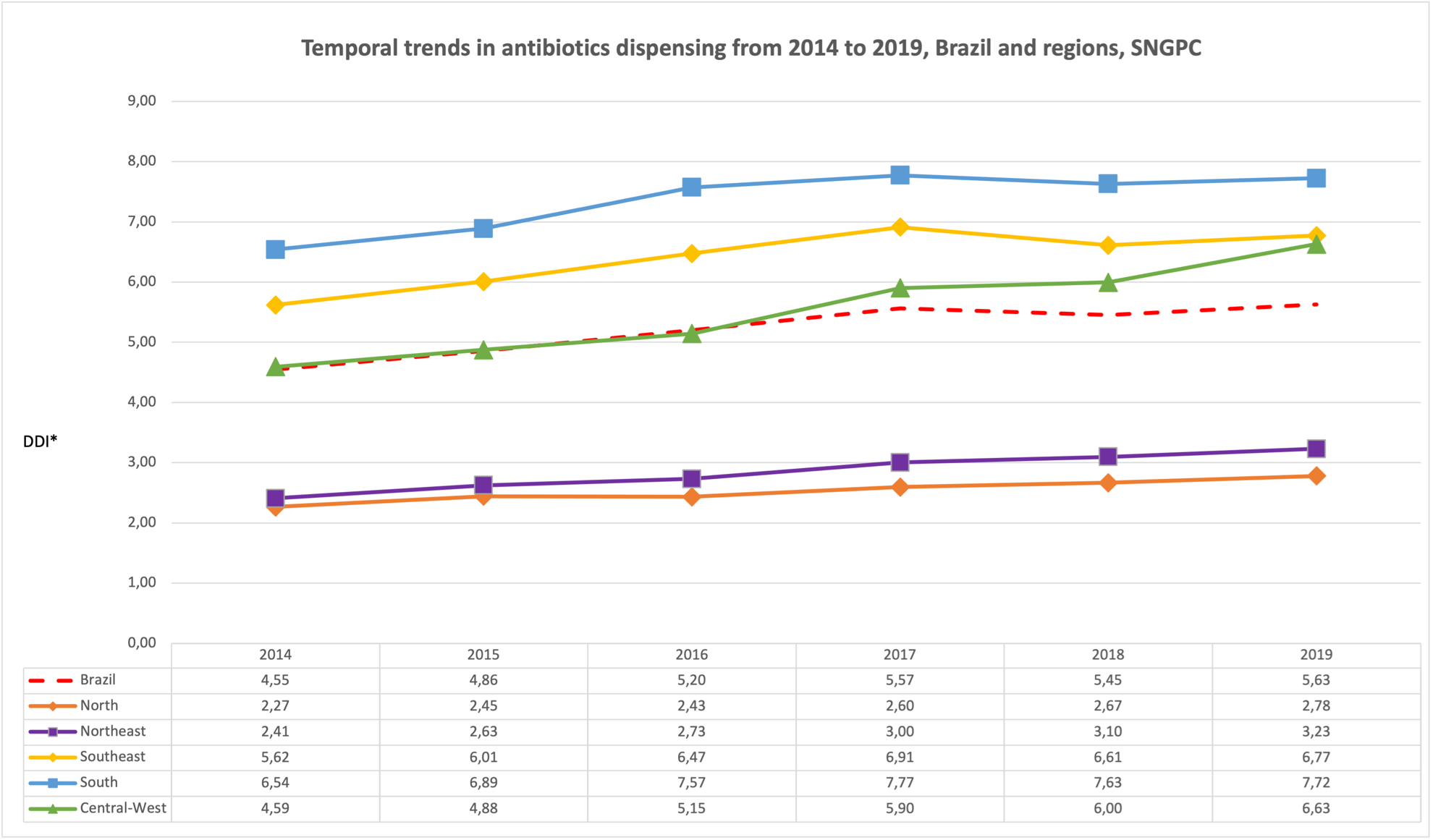

Supplement: Supplementary file 1 — Supplementary Material 1 [file 13756_2024_1412_MOESM1_ESM.tif]

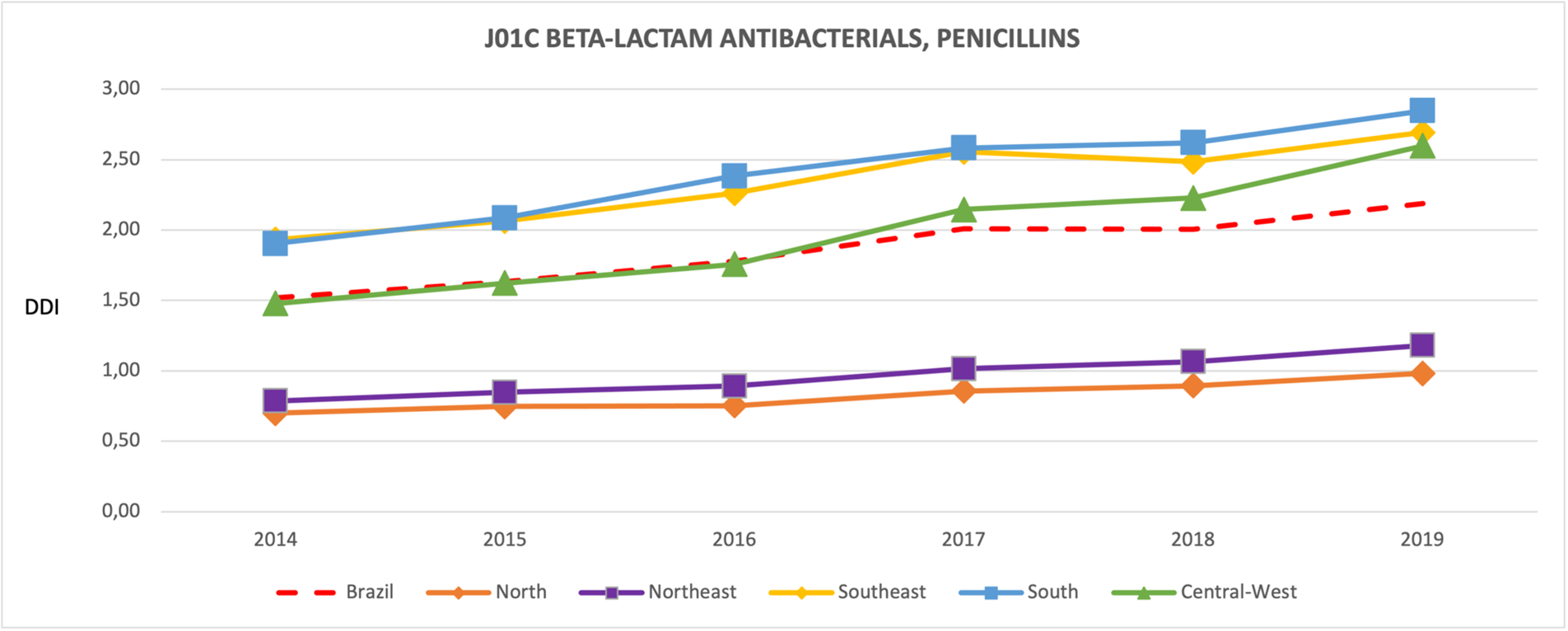

Supplement: Supplementary file 2 — Supplementary Material 2 [file 13756_2024_1412_MOESM2_ESM.tif]

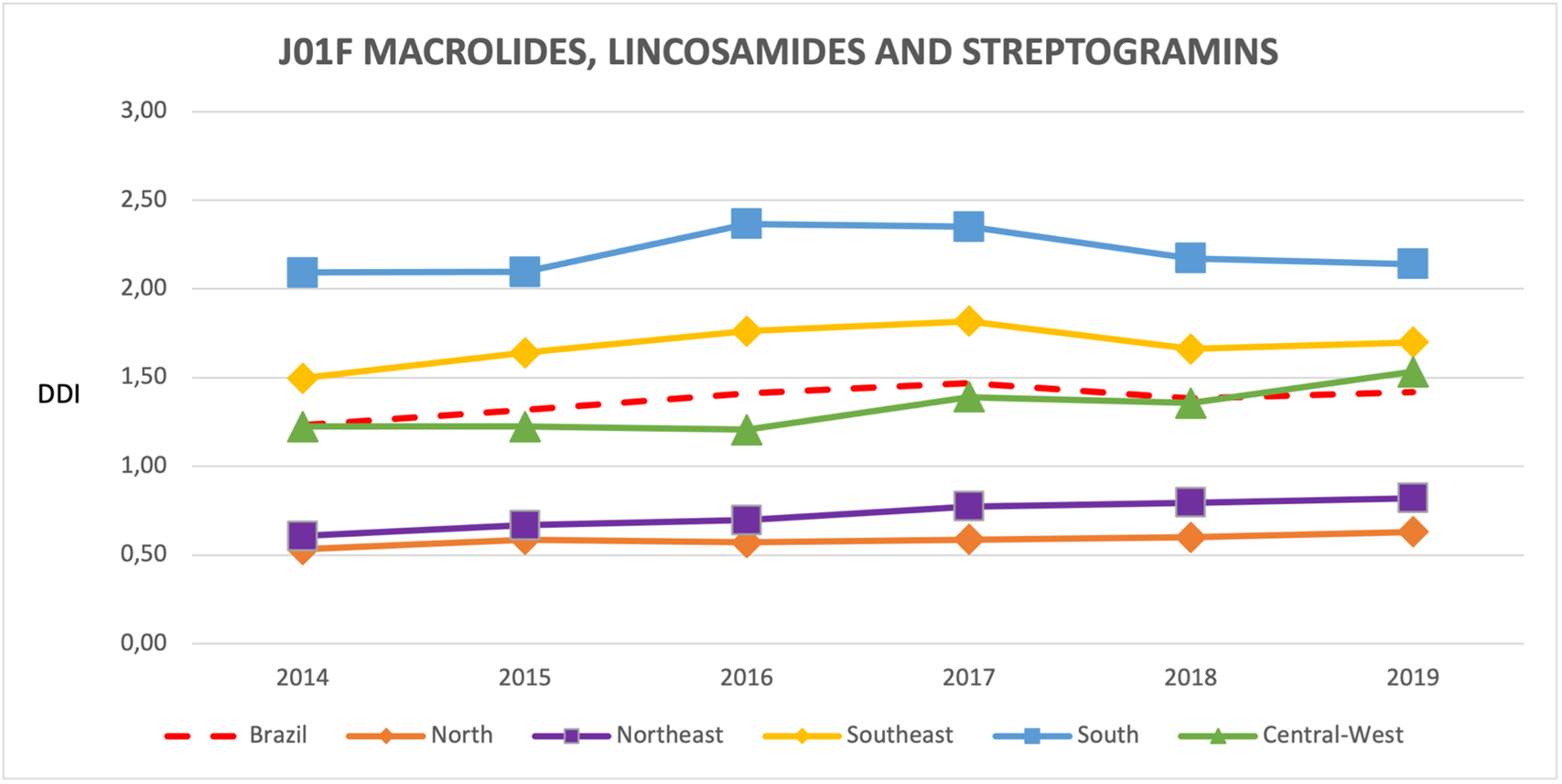

Supplement: Supplementary file 3 — Supplementary Material 3 [file 13756_2024_1412_MOESM3_ESM.tif]

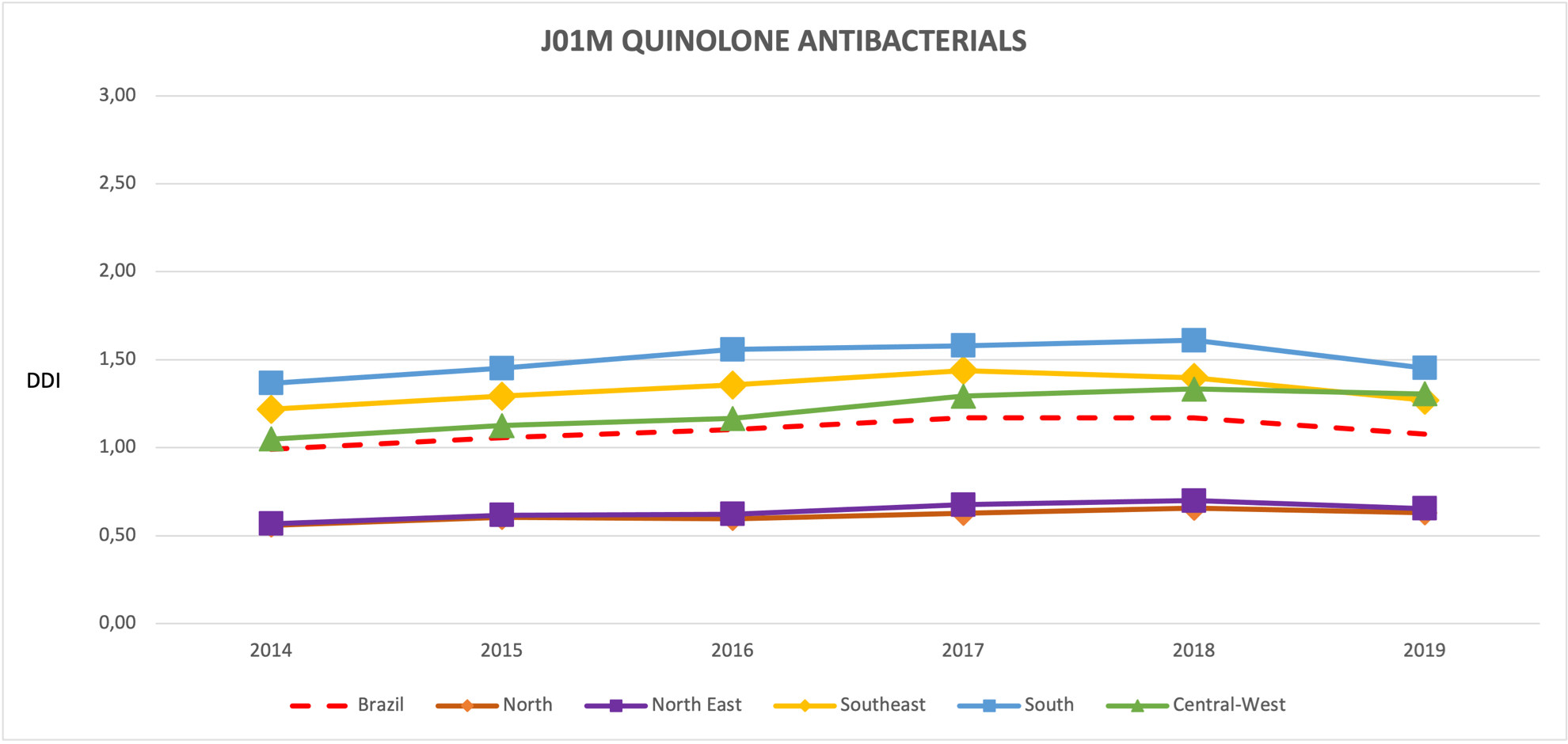

Supplement: Supplementary file 4 — Supplementary Material 4 [file 13756_2024_1412_MOESM4_ESM.tif]

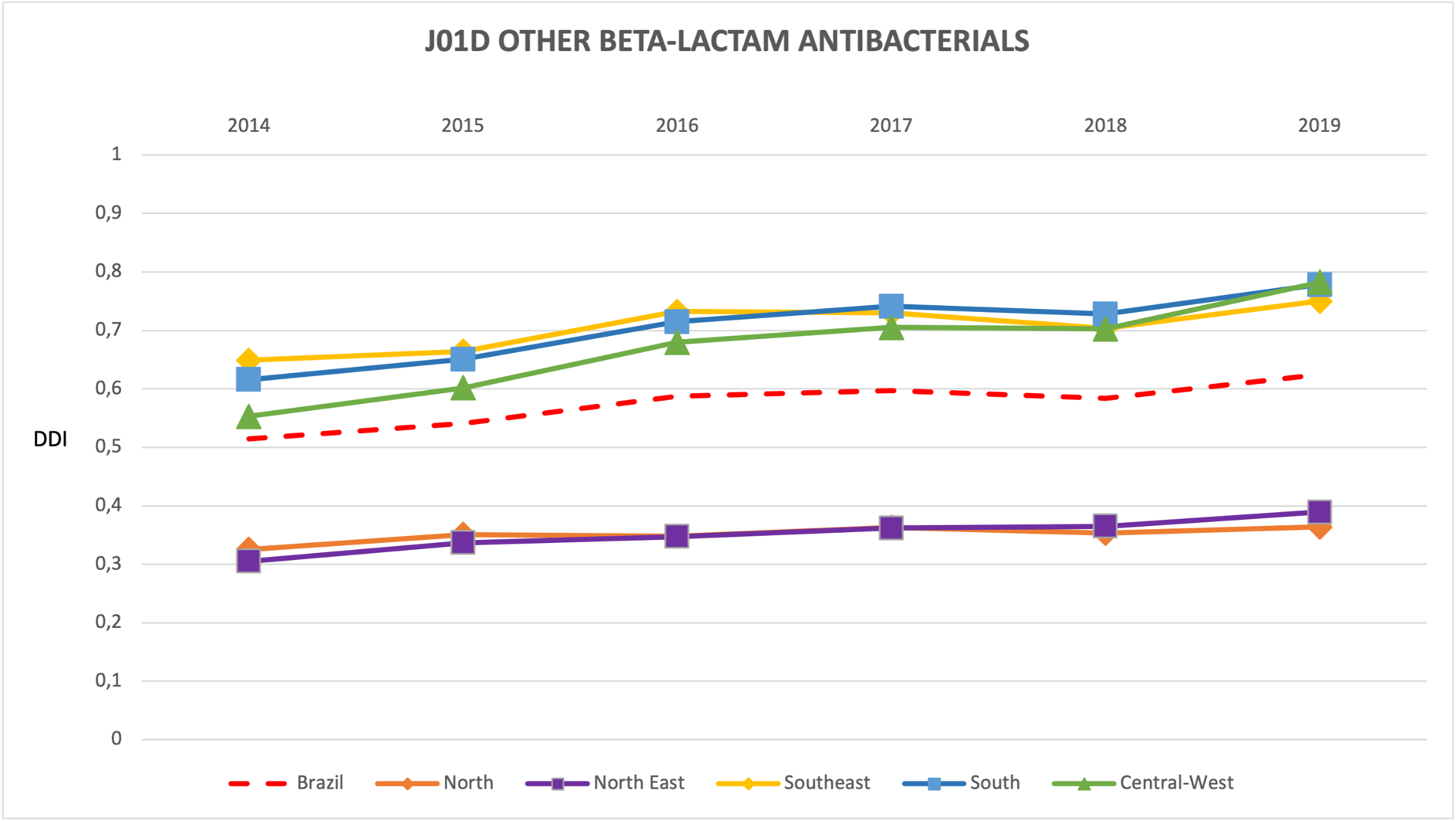

Supplement: Supplementary file 5 — Supplementary Material 5 [file 13756_2024_1412_MOESM5_ESM.tif]

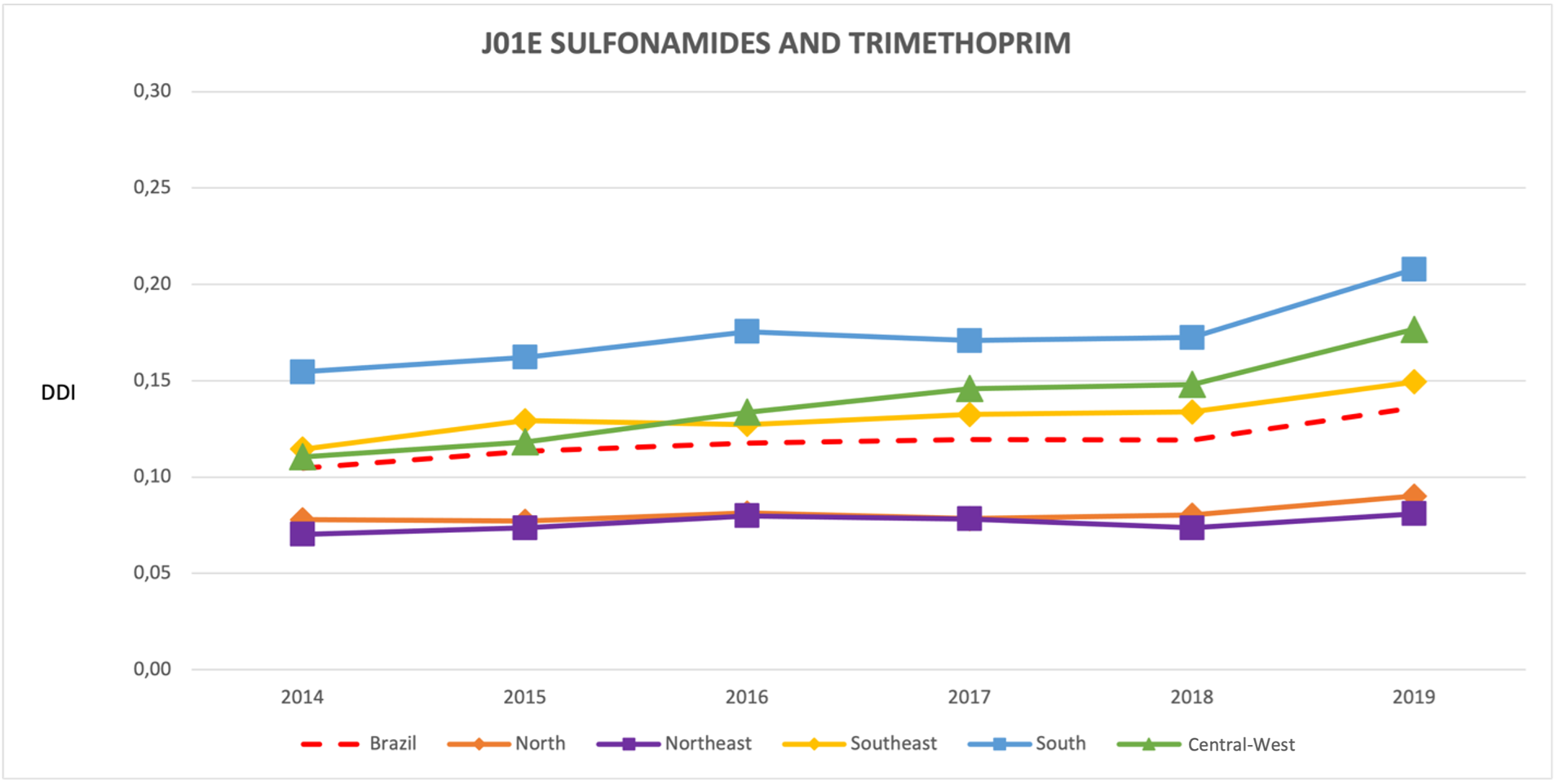

Supplement: Supplementary file 6 — Supplementary Material 6 [file 13756_2024_1412_MOESM6_ESM.tif]

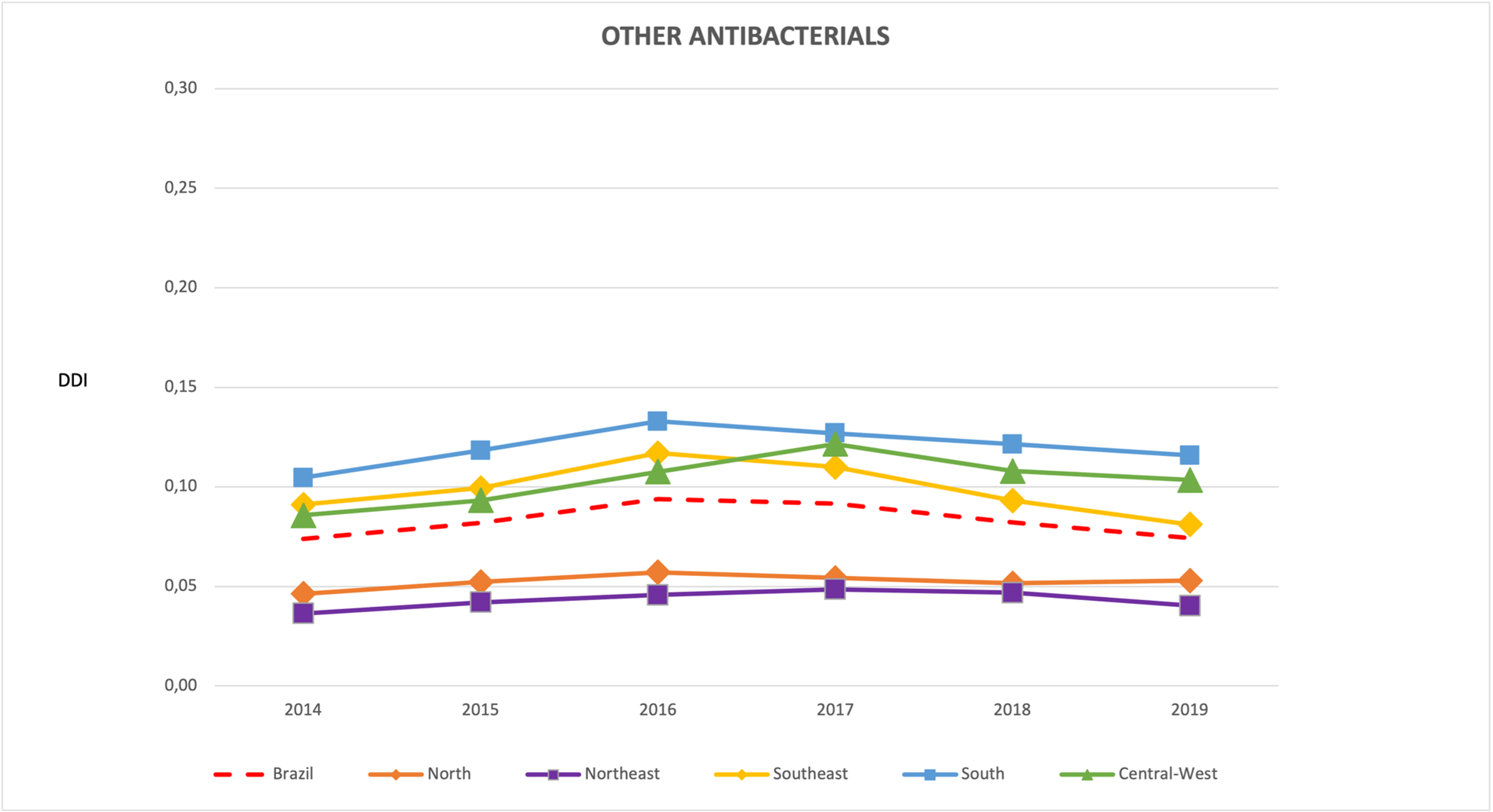

Supplement: Supplementary file 7 — Supplementary Material 7 [file 13756_2024_1412_MOESM7_ESM.tif]
