# Supplementary material for: Consumption of antibiotics in Brazil - an analysis of sales data between 2014 and 2019
Source: Antimicrob Resist Infect Control. 2024 Jun 9;13:60. doi: 10.1186/s13756-024-01412-6 (PMC11163732; doi:10.1186/s13756-024-01412-6)
Supplement: Supplementary file 8 — Supplementary Material 8 [file 13756_2024_1412_MOESM8_ESM.docx]

Additional File 1 - List of variables and definitions

| **Table S1. List of variables and definitions** | | |
| --- | --- | --- |
| **Variable** | **Variable in Database** | **Definition** |
| Year | ANO_VENDA | Year of sale of the medicine |
| Month | MÊS_VENDA | Month of sale of the medicine. |
| Federative Unit | UF_VENDA | Federative Unit of the address of the pharmacy or drugstore, registered in Anvisa's database, representing the UF where the sale took place. |
| Active ingredient | PRINCIPIO_ATIVO | Name of the active ingredient of the industrialized medicine, as registered in the medicine registration, in the Anvisa database. When a drug has more than one active ingredient, each one is separated by the “+” character. Ex.: “ACTIVE ingredient 1 + ACTIVE ingredient 2 |
| package | DESCRICAO_APRESENTACAO | A Drug Presentation represents the way a drug is presented on the package.  . |
| Number of sales | QTD_VENDA | Number of sales |
| quantity of medicine boxes or bottles | QTD_VENDIDA | Sold quantity of medicine boxes or bottles. |
| Council of the professional | CONSELHO_PRESCRITOR | Class Council of the professional who prescribed the medicine sold. |
