# Supplementary material for: Consumption of antibiotics in Brazil - an analysis of sales data between 2014 and 2019
Source: Antimicrob Resist Infect Control. 2024 Jun 9;13:60. doi: 10.1186/s13756-024-01412-6 (PMC11163732; doi:10.1186/s13756-024-01412-6)
Supplement: Supplementary file 9 — Supplementary Material 9 [file 13756_2024_1412_MOESM9_ESM.docx]

| **Table S2. Sales volume by region in the years 2014 and 2019.** | | | | | | |
| --- | --- | --- | --- | --- | --- | --- |
|  | **2014** | | **2019** | | **Difference between**  **2014 and 2019** | |
|  | **Number of Sales** | **Percentage of Number of Sales (%)** | **Number of Sales** | **Percentage of number of sales (%)** | **% Number**  **of Sales** | **Percentage of national number of sales** |
| **Brazil** | 44,964,792 | 100.0 | 59,319,550 | 100.0 | + 31.2 | - |
| **North** | 2,237,617 | 5.0 | 2,795,646 | 4.7 | + 24.3 | - 0.3 |
| **Northeast** | 7,371,755 | 16.4 | 9,466,654 | 16.0 | + 28.4 | - 0.4 |
| **Southeast** | 23,219,570 | 51.6 | 30,087,600 | 50.7 | + 29.6 | - 0.9 |
| **South** | 8,584,818 | 19.1 | 11,451,390 | 19.3 | + 33.4 | + 0.2 |
| **Central-West** | 3,551,027 | 7.9 | 5,518,253 | 9.3 | + 55.4 | +1.4 |
|  | | | | | | |

- **Population size IBGE (2023): North 17,349, 619 (8.5%); Northeast 54, 644, 582 (26.9%); Southeast 84, 847, 187 (41.8%); South 29, 933, 315 (14.7%) and Central-West 16,287,809 (8 %)**
